# Medical Miscellanies

**Published:** 1817-09

**Authors:** 


### PART IV.

### MEDICAL MISCELLANIES.

### THE PORTE FEUILLE, No. VIII.

OR,

DEPOT FOR DETACHED FACTS AND INTERESTING PHÆNOMENA OCCURRING IN THE PRACTICE OF THE FRIENDS OF THE MEDICO-CHIRURGICAL JOURNAL.

"Ore trahit quodcunque potest atque addit acervo."

### *On the Efficacy of the Cold Affusion in cutting short the Paroxysm of certain Convulsive Diseases.*

(Communicated in a Letter to Dr. Johnson.)

Some five years ago, in the middle of summer, I was requested to visit a young woman, between 20 and 25 years of age, who, for several days, had lain in strong convulsions. She was unmarried, possessed an interesting exterior, and all the physiognomical traces of exquisite sensibility. Her health had long been precarious; her menstruation regular, but defective in quantity; her bowels habitually costive. She had been for some time under the influence of depressing moral affections, and had recently suffered, with the rest of the family, from severe epidemic ophthalmia.

On my first visit, about noon, I found the young woman in bed, perfectly still and apparently insensible. She would not answer any question nor shew her tongue. When an attempt was made to separate the eye-lids, she drew back with a sort of fretful impatience, and immediately closed them. The temperature of the skin was natural; the pulse wholly undisturbed. There was neither hardness nor tension of the abdomen; but pressure on the epigastric and umbilical regions was evidently productive of uneasiness. I learned from the attendants that she had taken but little nutriment, liquid or solid, from the beginning of her illness; that during the greater part of the preceding night, she had been in such strong convulsions as to require five or six stout women to retain her in bed, and much apprehension had been felt of her dying in them. The convulsive paroxysm, it appeared, was ushered in by an undulatory motion and distension of the throat, probably a species of *globus hystericus*. It continued, without intermission, from three quarters of an hour to an hour; and the interval, rarely of such long duration, was passed in the state of lethargic tranquillity which I have described. The bowels had been very freely evacuated by active mercurial and aloetic purgatives, without sensible relief. Of the condition of the alvine and urinary discharges, nothing satisfactory could be learned.

I staid with my patient more than an hour, in the hope of witnessing a paroxysm; but none recurred. I then gave directions respecting the use of the cold affusion in the event of their return, and had left the house on my way home, when a messenger came after me with the intelligence that the fits were beginning again. I got back just in time to see the commencement of the paroxysm, and a curious spectacle it was. The superior and inferior extremities, after being stretched out for about two minutes, in inflexible rigidity, were suddenly retracted towards the body. Then, the young woman lying completely supine, the head and feet began insensibly to approach each other, till the trunk formed a tall arch, supported by the head and feet; narrower at the base than the summit, the external boundary of which was the abdomen, and in fact, precisely resembling in figure an ass-shoe set upright. The countenance, meanwhile, was dreadfully contorted. The attempt to change this singular position was difficult from the general rigidity of the body; but, when it was persisted in, the arch sank, and was exchanged for a rapid winding motion, which I could only compare to the writhing of a wounded eel just taken from the water. Finding all efforts at restraint useless, I directed my patient to be removed from the bed just as she was, and laid upon a small sheet, supported at each corner by a strong female. Two buckets of water, cold from the pump, were set beside me. One of these I dashed on the young woman just at the height of the paroxysm. The shock was terrible; she gasped, and sank backward, apparently lifeless, on the sheet. After a few moments, a slight convulsive motion of the limbs was observed, andI had recourse to the bucket of reserve; this completed the business.

The body of my patient was then wiped quite dry with coarse towels, and replaced in a warm bed, with bottles of hot water applied to the feet. I remained in the room half an hour. By that time, a genial warmth, succeeded by fine perspiration, had become universally diffused. The young woman appeared to have fallen into a tranquil slumber, unbroken by the slightest trace of convulsion. On my departure, I strictly enjoined an immediate repetition of the cold affusion in the event of a relapse: and the attendants, highly pleased with the control which they had unexpectedly acquired by this rough remedy, over a frightful and harassing complaint, promised steady adherence to my directions.

Towards evening, a messenger came to inform me that the convulsions had returned very slightly about four o'clock, but had completely subsided on re-employment of the water; and that the young woman had since left her bed, and taken tea very comfortably with the family, to their great joy and surprize. I prescribed a continuance of the purgative plan; and during several months which I sojourned in that part of the country, the convulsions never re-appeared.

In this case, the source of the disease had evidently been, in great measure, removed by the operation of the purgatives, ere the cold affusion was resorted to. Thus, to break through the chain of diseased actions, which, by a peculiar law of the animal œconomy, often outlive their cause, and become independent of it, was alone necessary; and this the cold affusion most effectually accomplished. But to the fulfilment of such indication, the exhibition of this powerful remedy is not exclusively restricted. I have more than once used it with signal benefit to gain a truce for the employment of the lancet, and internal means, in severe and protracted cases of epilepsy and other convulsive affections, where, from the violence of the patient's struggles, so long as the paroxysm lasted, the institution of any remedial plan must have been utterly impracticable.

S.

### *Cicatrization of the Vagina.*

Mrs. B. after her second labour, (which was tedious, and during which, much violence was used by a woman calling herself a midwife) could never retain her urine, which constantly dribbled from her, except when in a recumbent position. She also felt "such soreness in her womb," to use her own expression, as prevented her walking for many months after confinement, without great pain and uneasiness.

She suckled the child nearly fourteen months, weaned it; menstruated six weeks after, and became pregnant again.

At the labour of her third child, after a number of strong pains, an examination being made, the vagina was found to terminate, at about an inch from the os externum, in a firm cicatrix, or adhesion, having a cartilaginous feel, and forming, as it were a septum, between the cavity of the uterus and the vagina, so completely impervious, that the point of a probe could no where be passed through.

During four hours, the pains being very frequent and strong, the head made no advance; evidently in consequence of the resistance afforded by the cicatrix, against which it was propelled by each effort of the uterus with considerable force.

A bistoury, guarded by the finger, was passed through the os externum, and an aperture scratched in the cicatrix of a sufficient size to admit the point of the index finger of the left hand, which served as a director to a probe-pointed bistoury in enlarging the incision on each side, to the extent of nearly half an inch. In doing this, the membranes were cut, and liquor amnii discharged.

The labour now proceeded in a perfectly natural manner; the *artificial* opening gradually dilated by the pressure of the head, and in two hours the child was born. No hæmorrhage, unusual soreness, or any unfavourable occurrence followed the labour.

The cicatrix when divided, felt rather more than a third of an inch in thickness; and the bistoury, while cutting it, communicated a sensation to the fingers similar to that which is felt on dividing tendinous fibres. G. W.

---

### *Case of distressing Head-ache.*

A young woman had been afflicted with most distressing head-ache, occasionally aggravated, for years. The pain was situated in one of the frontal sinuses, apparently, and always fixed. A few days ago, a piece of calcareous concretion, the size of a small hazel nut, came away while blowing the nose, and the head-ache has dissappeared. L.

---

### MISCELLANIES.

Quicquid agunt Homines.

### *To the Editors of the Medico-Chirurgical Journal & Review.*

Gentlemen,

In the 20th Number of your Journal, there are several Cases which well deserve the attention of the observant physician and pathologist. The train of "*conversion of diseases*," so well related by Dr. Felix, is eminently interesting; though I should be much inclined to substitute *conversions of disease*, for "conversion of diseases, since the various phænomena were probably only different manifestations of the same malady.

—— Facies non omnibus una

*Nec diversa* tamen.

Dr. Kinglake, indeed, on perusing Dr. Felix's remonstrances against exposing the "rosy feet" of the old gentleman to cold, may exclaim, "O ego non Felix, quem tu fugis," &c. Yet, there is good reason to catenate the exposure and retrocession, as *cause and effect* in this, as well as inmany other cases; and the impartial and unbiassed medical philosopher will not fail to bear this memorable example in mind. In respect to the sudden and spontaneous disappearance of hydrocele, I beg leave to observe, that Pott witnessed a similar metastasis on the accession of a gouty attack in the feet; and that Guilbert, a French physician, did the same: "Nous avons vu aussi la guêrison rapide d'un hydrocèle, chez un homme sujet a divers accidens de la goutte fibreuse, sans operation chirurgicale." *Dictionnaire de Sciences Medicales*, tom. xix. p. 134.

And now that we are on the subject of gout, I beg to congratulate Dr. Johnson on his wisdom in declining any contest with Dr. Balfour; for although the latter gentleman asserts, that his "compression and percussion are *conciliatory*" (p. 168), yet I should not care to wrestle with a man of his *striking propensities*, or to question the effect of an "application of percussion, *a capite ad calcem*," on his revulsive principles.

There is a most interesting case in the same Number of your Journal, p. 154, where Corvisart himself was deceived respecting a *supposed* aneurism of the heart. Without at all questioning the ingenuity of the editorial remarks, or those of Dr. Duchateau, on this case, I would venture to surmise, that the preternatural action of the heart "elevating the parietes of the chest by frightful pulsations," was not entirely "sympathetic of epigastric derangement," but a morbid action going on in these parts (probably gouty affection); which morbid action being suddenly transferred to the abdomen, there produced inflammation and rapid gangrene of the colon.* I do not see any of those marks which characterize *chronic* inflammation in the omentum or intestines; and that misplaced gout will produce affections of the heart and lungs, imitating angina pectoris and asthma, we have many authorities. *Vide* Article "*Goutte*," in the French *Dictionnaire des Sciences Medicales.*

Before parting, may I be permitted to suggest an improvement on a note in the 131st page of your last Journal. While I admire the spirit and critical acumen of the Reviewer of the pamphlets of Spurzheim and Gordon, I am free to confess, that even the semblance of a pun on the melancholy occasion of Dr. Parry's illness, appears to me somewhat hazardous. Nevertheless, the compliment ("if in our ashes live their wonted fires"), cannot be displeasing to either the dead or the living. I therefore propose the following alteration: Instead of "quando ullum invenient *Parem?*" I would say, Eheu! quamdiu altero careamus *Pari?* or, Quando ullo potiemur *Pari?*

MEDICUS.

*12th August*, 1817.

---

* Gouty action will be, in one viscus or part of the body, a *neuralgia;* and in another, a real *phlegmasia.* We need not therefore wonder that no trace of inflammatory disorganization could be perceived in the chest, while the most dreadful effects of it were visible in the abdomen. M.

On the 1st of August, 1817, the *Senatus Academicus* of the University of Edinburgh conferred the Degree of *Doctor in Medicine* on the following gentlemen, after having gone through the appointed examinations,* and publicly defended their respective inaugural dissertations :

FROM ENGLAND.

| | | |
|---|---|---|
| Charles E. Bacon - - - - | Thesis De | Typho |
| Thomas Barnes - - - - | —— | Erysipelate |
| John Grove - - - - - | —— | Concoctione Prava |
| Samuel Hibbert - - - - | —— | Vita Humana |
| William Kettle - - - - | —— | Dysenteria |
| Martin Loy - - - - - | —— | Enteritide |
| Edward T. Luscombe, Surg. 34th Regiment - - - - | —— | Sanitate Militum conservanda |
| Thomas Norris, Surgeon 2d Lancashire Militia - - - | —— | Regimine |
| John Ord - - - - - - - | —— | Phthisi Pulmonali |
| Benjamin C. Pierce - - - | —— | Bronchocele |
| Robert Smith - - - - - | —— | Febre Infantili Remittente |
| Joseph Stephenson - - - | —— | Humani Generis Varietatibus |
| William Thomson - - - | —— | Neuralgia |
| William B. Thornton - - | —— | Febre Puerperarum |
| Christopher Vickers - - - | —— | Hypochondriasi |
| Charles G. Welchman - - | —— | Apoplexia Sanguinea |
| Joseph Widdup, Dispenser to the Naval Hospital - - | —— | Iritide |
| James T. Young - - - - | —— | Trismo Nascentium |

FROM SCOTLAND.

| | | |
|---|---|---|
| William Bain - - - - - | Thesis De | Vulneribus |
| Jo. Bell - - - - - - - | —— | Dyspepsia |
| Jo. Boggie - - - - - - | —— | Hydrope Anasarca |
| William Boyd, Surg. R. N. | —— | Febre Minorcæ |
| William Burrell - - - - | —— | Ophthalmia |
| R. Carnegy - - - - - | —— | Ulcusculis Venereis |
| David Chalmers - - - - | —— | Febre continua contag. |
| G. S. Charters - - - - | —— | Vi Animi |
| James Clark, Surgeon, R. N. | —— | Frigoris Effectibus |
| Thomas Craig - - - - - | —— | Delirio |
| William Crawford - - - | —— | Apoplexia |
| Andrew Dods, Surg. R. N. - | —— | Febre Mediterranei |
| James D. Flemming - - - | —— | Hernia |
| John Forbes, Surgeon, R. N. | —— | Mentis Exercitatione et felicitate exinde deriv. |

* For these "appointed examinations," see our Journal for June 1817, vol. iii. p. 524.

| | | |
|---|---|---|
| James Gellatly - - - - | Thesis De | Hernia |
| D. Gibson - - - - - - - | —— | Diabete |
| John Gillies, Surgeon, R. N. | —— | Iritide |
| James Guthrie, Surg. R. N. | —— | Cholera |
| Robert Hogg - - - - - - | —— | Ictero |
| James Inglis - - - - - - | —— | Hydrocephalo |
| Thomas Inglis - - - - - - | —— | Pertussi |
| P. Lamond - - - - - - - | —— | Hepatitide |
| Jo. Locke - - - - - - - | —— | Cerebri Compressione |
| William Lucas - - - - - | —— | Morborum Diagnosi |
| Rob. M'Dowall, Surg. R. N. | —— | Dysenteria |
| William Montgomerie - - | —— | Hepatitide |
| James Montgomery - - - | —— | Phthisi Pulmonali |
| Walter Oudney, Surg. R. N. | —— | Dysenteria Orientali |
| G. M. Paterson - - - - | —— | Mania |
| D. Ramsay - - - - - - | —— | Angina Pectoris |
| Archib. Robertson, Surgeon, R. N. - - - - - - - | —— | Dysenteria Regionum Calidarum |
| Ebenez. Scott, Surgeon, R. N. | —— | Rubeola |
| Jo. Squair - - - - - - - | —— | Hydrocephalo |
| William Taylor - - - - | —— | Apoplexia |
| Robert Tod, Surgeon, 52d Regiment - - - - - - | —— | Femoris Amputatione in Cavitate Cotyloidea |
| David Wright - - - - - | —— | Hæmoptysi |
| Thos. Braidwood Wilson, Surgeon, R. N. - - - - - - | —— | Hepatitide |

FROM IRELAND.

| | | |
|---|---|---|
| Robert Barlow, Surgeon, West Meath Militia - - - - | Thesis De | Apoplexia Sanguinea |
| John Bernard, Surgeon, R. N. | —— | Dysenteria |
| Tho. Bulkley, Surg. of — Reg. | —— | Enteritide |
| M. O. Busteed - - - - - - | —— | Phrenitide |
| Daniel Cantillon - - - - - | —— | Cholera |
| Rob. Alex. Chermside, Surg. 10th Dragoons - - - - | —— | Aqua frigida in Febrib. |
| J. J. Cronin - - - - - - | —— | Hepatitide |
| M. Devitt - - - - - - - | —— | Febre Intermittente |
| Henry Gardner - - - - | —— | Asthmate |
| J. Gernon - - - - - - - | —— | Ictero |
| George Herrick - - - - | —— | Aneurismate |
| James Hunter - - - - - | —— | Inflammatione |
| James T. Hurst - - - - | —— | Aneurismate |
| James Kelly - - - - - - | —— | Hepatitide |
| Sam. Kenning, Surg. of Ordnance | —— | Febre Bulamense |
| Nicolas King - - - - - | —— | Submersione |
| Jer. Leyne - - - - - - - | —— | Dyspepsia |
| Jos. Little - - - - - - - | —— | Ictero |
| Philip Lyons - - - - - - | —— | Hepatitide |
| William Magill - - - - | —— | Tetano |

| | | |
|---|---|---|
| Thomas M'Keever - - - | Thesis De | Aqua |
| Ed. Mollony - - - - - - | —— | Rubeola |
| William Nason - - - - - | —— | Pertussi |
| Alexander J. Nicholson - - | —— | Intemperantia |
| Lawrence O'Reilly - - - | —— | Aquæ frigidæ usu |
| John Peebles - - - - - - | —— | Ischuria |
| Stephen Ronan - - - - - | —— | Fungo Hæmatode |
| John Short, Surg. of — Reg. | —— | Febre Flava Hispan. |
| Samuel Sinclair, Surg. R. N. | —— | Cœlo Maris Mediterranei |
| Robert Stephenson - - - | —— | Respiratione |
| And. Thomson, Surg. of — Reg. | —— | Vulneribus Scloppeticis |
| Matt. Walsh - - - - - - | —— | Rubeola |

FROM JAMAICA.

| | | |
|---|---|---|
| William Rhodes Bernard - | Thesis De | Calore Animali |
| David Shaw - - - - - - | —— | Chorea |
| Henry V. Towton - - - | —— | Somno et insomniis |

FROM BARBADOES.

| | | |
|---|---|---|
| N. L. Young - - - - - - | Thesis De | Cutis Inhalatione |

FROM HAMBURG.

| | | |
|---|---|---|
| Amicus Bouè - - - - - - | Thesis De | Methodo *Floram* conducendi |

The following is a copy of the *Diploma:*

" Nos Academiæ Jacobi VI. Scotorum Regis, quæ Edinburgi est, Primarius cæterique Professores hoc scripto testatum volumus A—— B——, postquam se suosque in Re Medica Progressus Facultati Medicæ probâsset, luculento testimonio ab ea nobis commendatum, summos in Medicina honores, Gradum nempe Doctoralem (subjecta prius publicæ Professorum Censuræ Dissertatione sua de ——— ———, delato Jurejurando, solennibusque rite peractis,) consecutum esse; eique amplissimam Potestatem Medicinam ubique Gentium, legendi, docendi, faciendi, concessam, aliaque omnia Privilegia, Immunitates, Jura, quæ hic, aut usquam alibi, ad Doctoratûs apicem evectis concedi solent. Cujus Rei quò major esset fides, Nos, sigillo communi Academiæ appensæ, Chirographa apposuimus, Edinburgi Anno Salutis Humanæ millesimo octingentesimo, &c. mensis Augusti die primo."

Here follows the Oath, or *Sponsio Academica,* above referred to, administered to Candidates taking the Degree of M. D.

" Ego A—— B——, Doctoratus in arte Medica titulo jam donandus, sancte coram Deo, cordium scrutatore, spondeo, me in omnibus grati animi officiis erga Academiam Edinburgenam ad extremum vitæ halitum perseveraturum: Tum porro artem Medicam caute, caste, et probe exercitaturum, et, quoad potero, omnia ad ægrotorum corporum salutem conducentia, cum fide procuraturum; quæ denique inter medendum visa vel audita sileri conveniat, non, sine gravi causâ, vulgaturum. Ita presens spondenti adsit Numen!"

*N. B.* Should any of the Candidates be Quakers, as their religious principles prevent them from swearing in the customary manner, a Declaration, to the same purpose, is tendered them to sign.

### *Regulations respecting Degrees in Medicine in the University of Glasgow.*

The following Regulations, respecting Degrees in Medicine, were unanimously enacted into law, by the Senate of the University of Glasgow, on Saturday, the 12th of December, 1812. (The Operation of them commenced on November 1, 1813.)

I. Before any person can be allowed to be a candidate for a degree in Medicine, in this University, he shall appear, personally, before the Senate; and lay before them satisfactory evidence, that he is not under twenty-one years of age.

II. He shall produce evidence, as above, that he has during at least three years, or sessions of six months each, regularly attended the following medical classes in some University or Universities; or two sessions, if he have studied, during other two years, under eminent medical teachers in London; viz. Anatomy and surgery, during three sessions; the theory and practice of physic, during two sessions; chemistry, during two sessions; materia medica and pharmacy, during two sessions; or one session, if he have attended an apothecary's shop, during two or more years; midwifery, during one session; and botany, during one course.

III. He shall bring forward evidence, that during one year, at least, he has attended medical classes in this University.

IV. The candidate shall undergo three examinations, in private, by the Medical Professors of the University; and write a Commentary on an aphorism of Hippocrates, and another on a case of disease, propounded to him by the said Examiners. The first examination shall be on anatomy and physiology; the second on the institutions and practice of physic; and the third on chemistry, materia medica, and pharmacy.

V. The Examiners shall report to the Senate their opinion respecting the medical knowledge of the candidate; and, if their report be favourable, his name, as a candidate for a degree, shall be entered on the minutes of the Senate; and a day shall be fixed when the candidate shall read his Commentaries on the aphorism and case, and answer such questions, on the several branches of medical science, as shall be put to him by the Examiners, in presence of the Senate. If the Senate be of opinion that the candidate has shown himself worthy of a degree, it shall be conferred, in presence of the Senate, by the Vice-Chancellor, provided the candidate have not published a Thesis, which he may or may not do, according to his own option; but, if he have published a Thesis, he must defend it, and the degree must be conferred in presence of the students and other members of the University, assembled by Program in the Comitia.

VI. The whole of the examinations shall be carried on, and the Commentaries on the aphorism and case, must be written in the Latin language.

---

The occurrence of Small-pox in persons who had been previously vaccinated, appears to have been of late very frequent in York. In all the cases, however, the eruption has been so modified in its appearance, and has subsided so early, as to have excited considerable doubt in the minds of many practitioners, whether this anomalous disease were really small-pox. The question was made a subject of experiment by Dr. Baldwin Wake, of that city. From a pustule in a boy, who had been vaccinated some years before, and who was then affected with this modified eruption, he inoculated a subject who had never been vaccinated, in whom the inoculated disease exhibited all the genuine characters of small-pox, and went through the regular stages of that disorder; thus affording a convincing proof of the protecting power of vaccination against the full effects of variolous infection. To account for the occurrence of small-pox in vaccinated patients, Dr. Wake supposes the system not to have been saturated with vaccine virus; and he recommends an invariable adoption of Mr. Bryce's method of testing by a subsequent insertion of vaccine virus during the progress of the first pustule; and Dr. W. asserts, that in no one person thus treated, has he ever known the small-pox to appear even in its mildest modified form.

ROYAL COLLEGE OF SURGEONS.

*Jacksonian Prize.*—The Prize for the year 1814, and that for the year 1816, not having been adjudicated, three Prize Subjects are proposed for the Year 1818, viz.—" Scrofula—Inflammation —Diseases of the Skin."—The Prize Subject for the present year, 1817, is " Deafness, and the Diseases and Injuries of the Organ of Hearing." Dissertations upon which must be delivered at the College before Christmas Day next.

---

*At a Meeting of the Associated Apothecaries and Surgeon-Apothecaries of England and Wales, held, by public Advertisement, at the Crown and Anchor Tavern, August the 20th,* 1817,

The Eighth Report of the General Committee was read, as follows:

Circumstances having rendered it expedient to convene a general meeting of the members of the Association, its Committee begs leave to detail the events which have occurred since the publication of their last Report, dated 24th of April, 1816.

By a reference to that document, it will be seen what steps the Committee has taken to procure such alterations as appeared advisable in the Bill intended to be brought before Parliament by the College of Surgeons during that session; and als an amendment of the Apothecaries' Act. The Royal College of Surgeons did not, however, then introduce any Bill. But the Court of Assistants of the society of Apothecaries, notwithstanding the declaration of its Bill-Committee, "that such practical inconvenience had not arisen from the alleged defects of the Act, as to induce it to recommend to the Court of Assistants any immediate application to Parliament,' did immediately afterwards intimate, that as the Secretary at War, Lord Palmerston, was about to bring in a Bill to amend the Act, as far as it affected army and navy medical officers, the Court meant to embrace an obliging offer of his Lordship, of making such other alterations in the Act as would correct its defects, &c.; and Dr. Burrows and Mr. Field met the Bill-Committee at the Hall, to discuss the points which were stated as requiring amendment. This intention of the society was made known to your Committee, and some Resolutions relative to the subject were passed on the 15th of May. But the session closed; and the amending Bill was not brought forward, either by Lord Palmerston or by the Society.

In January last, the Chairman received a copy of a new Bill, which the College of Surgeons had arranged. This was submitted to the Committee on the 21st of that month. The Committee approved this Bill, as far as it related to the practice of surgery, but reiterated its objection to the amount of the fees for diplomas as being too large; considering that by the Act every person, henceforward practising surgery, would be compelled to apply for a diploma; whereas, under present circumstances, his coming before the College is entirely optional. A request was consequently transmitted to the College, that it would re-consider the subject of fees; accompanied with a copy of the following Resolution: viz. "That this Committee anxiously hope that the Royal College of Surgeons will not neglect the opportunities allowed by the intended Bill, of examining into the qualifications of candidates in the knowledge of Midwifery."

This Bill was expected to be introduced early in the last session of Parliament; but that has also passed without any application being made.

Five years have elapsed since your Committee was appointed; during which, it has been reduced by the death of many valuable members, by secessions and other causes, from forty-five to about twelve or fourteen effective persons;—a number by far too few to perform the ordinary business of the Association, or to constitute a due representation of the great body of general practitioners.

The intended resignation of the Chairman, Dr. Burrows, is a circumstance which your Committee has most sincerely to lament; for in him, from the length of time he has so honourably and zealously laboured in that very ostensible and important situation, all the most material details of the business have centered. His retirement imposes the obligation of appointing a successor. It would be an act of injustice to Dr. Burrows, if the Committee withheld from the Association the fact, that so long ago as May, 1816, he expressed, and for very sufficient reasons, his determination to retire from the chair; but, under the impression that the duties of the Committee would most likely be terminated with the last session of Parliament, he acceded to the general wish of the Committee, and was prevailed upon to extend his valuable services till that period. And although no application has been preferred to Parliament, either by the College of Surgeons or by the Society of Apothecaries, his anxiety and exertions have been unremitting to promote, as far as was in his power, the introduction of a Bill for the Regulation of Surgery, and the administration of the Apothecaries' Act, agreeably both to its letter and spirit.

By the subjoined statement of the funds of the Association, the Committee confidently trust it will appear, that a just regard has been paid to so important a resource; and although it may be deemed right to appropriate a portion of it for especial purposes at this meeting, yet it should ever be respected and preserved as a means applicable (when real occasion exists) to the support of the interests of the general practitioners in medicine. Nor can the Committee forbear expressing a hope, that the subscription book will still continue open, in order that the fund may be adequate to all possible exigencies.

The remaining members of your Committee have thought it a duty incumbent upon them to convene this General Meeting of the Association, that they may render to it an account of their proceedings; and with the utmost respect, they here surrender the trust which has been confided to them, and which they have endeavoured to execute faithfully and usefully.

*State of the Fund, May* 1, 1817.

| Dr. | £. | s. | d. | Cr. | £. | s. | d. |
|---|---|---|---|---|---|---|---|
| Law expences . . . . . | 144 | 9 | 9 | Balance of cash in hand at last Audit (Sept.3,1813) | 209 | 14 | 4 |
| Printing . . . . . . . . | 77 | 18 | 7 | Subscriptions since received | 146 | 5 | 1 |
| Advertising . . . . . . | 56 | 17 | 3 | By sale of the intended Bill | 7 | 7 | 7 |
| Sundry disbursements . | 43 | 14 | 4 | By return on advertisements | 3 | 13 | 6 |
| Meetings, public & Committee, during 4 years | 24 | 9 | 10 | By 4 years' interest on Exchequer Bill of £.1000 | 201 | 8 | 7 |
| | 347 | 9 | 9 | Balance £.220 19 4<br>Exchequer Bill £.1000 | 568 | 9 | 1 |

John Hunter, } Auditors.

R. S. Wells, }

Dr. Burrows having resigned the Chair, James Parkinson, Esq. was unanimously elected to fill that situation ; after which the following Resolutions were passed :

1. Resolved unanimously, That the most sincere thanks of this Association be given to Dr. Burrows for the indefatigable zeal, ability, and disinterestedness, with which he has filled the chair since the formation of the Association in 1812; and for the manly perseverance with which he met all the difficulties that were opposed to the passing of "An Act for the better regulating the Practice of Apothecaries," &c. ; by which the honour and respectability of the profession have been upheld, and the public interest most importantly served.

2. Resolved unanimously, That a purse of Five Hundred Guineas be presented to Dr. Burrows, the late chairman, as an acknowledgement of the services he has rendered to this Association, to the Profession, and to the Public.

3. Resolved unanimously, That the above Resolutions be advertised in the public papers.

4. Resolved unanimously, That the gentlemen who may this day be appointed Treasurers to this Association, be authorized forthwith to carry into effect the two preceding resolutions.

5. Resolved unanimously, That the sincere thanks of this Association be given to the members of the General Committee ; who, for a period of five years, have, with unwearied zeal, ability, and attention, devoted their valuable time to the interests of the Association, and the general improvement of the profession ; and who have consequently rendered an essential service to the public.

6. Resolved unanimously, That the thanks of this Association be given to the Treasurers for their services.

7. Resolved unanimously, That the Report of the General Committee, now read, be received and adopted.

8. Resolved unanimously, That this Association do continue permanent.

9. Resolved unanimously, That this Association determines to use its utmost exertions to promote the improvement of those branches of the Medical Profession exercised by general practitioners, and to protect their interests.

10. Resolved unanimously, That the interests of the Association be entrusted to a committee, with full powers to act according to its discretion.

11. Resolved unanimously, That the said committee shall consist of twenty-four members, resident in London and its vicinity; and that one moiety of them shall be members of the Society of Apothecaries, and the other moiety of non-members of that society.

12. Resolved unanimously, That Messrs. Hunter, Upton, Wells, Bowman, De Bruyn, Brande, Cates, Drew, Kerrison, Shillito, Seaton, and Malim, Members of the Society of Apothecaries—and that Messrs. Parkinson, Humby, A. T. Thomson, Cook, Haden, James, Reginald Williams, Hayes, Ogle, Nevill Wells, and Edward Leese, Non-members of the Society—do constitute the present committee.

13. Resolved unanimously, That the present Treasurers to this Association be desired, and are hereby authorised, to transfer to the Treasurers to be appointed at this meeting, the balance of the fund of this Association which may be now in their's, or the Banker's, Messrs. Gosling and Sharpe's hands.

14. Resolved unanimously, That the monies belonging to the Association be invested in public securities, bearing interest ; and be placed in the names of the three Treasurers now to be nominated, except such balance as is necessary to defray current expences.

15. Resolved unanimously, That Messrs. Parkinson, Hunter, and Upton, be Treasurers; and that the signature of two of the Treasurers be necessary to the disposal of any part of the fund.

16. Resolved unanimously, That, in order to establish a competent fund to preserve the interests of the Association, further subscriptions be received; and that any new Subscriber of One Guinea be enrolled as a Member of the Association.

17. Resolved unanimously, That subscriptions be received by any of the Treasurers, and by the Bankers to the Association, Messrs. Gosling and Sharpe, No. 19, Fleet Street.

18. Resolved unanimously, That the Committee be authorised by this General Meeting to receive from any member of this Association any well-authenticated case of illegal practising, for the purpose of submitting the same to the Court of Assistants of the Society of Apothecaries for their prosecution.

19. Resolved unanimously, That there shall be a General Meeting of the Association held annually, on the third Wednesday in July, at two o'clock; when any vacancy in the committee, or the office of Treasurer shall be filled up, the accounts of the current year be audited, and all other affairs relating to the Association shall be reported.

20. Resolved unanimously, That a fortnight's notice shall be given, by advertisement in the London Medical Journals, and in two morning and two evening newspapers, of the time and place of the annual meeting.

The Secretary being under the necessity of resigning—

21. Resolved unanimously, That the thanks of this meeting, with a purse of One Hundred Guineas, be presented to Mr. Ward, the late Secretary, as a mark of estimation for his services.

22. Resolved unanimously, That the Treasurers be requested forthwith to fulfill the object of this Resolution.

23. Resolved unanimously, That Mr. John Powell, of Newman Street, be appointed Secretary.

24. Resolved unanimously, That the thanks of this meeting be given to R. M. Kerrison, Esq. for the zeal displayed by him in his publications; as well as for the general interest he has taken to forward the objects of the Association.

25. Resolved, That a communication be made from this Association to the Court of Assistants of the Society of Apothecaries, begging them to ascertain and publish a list of those persons who were in practice up to the moment of the operation of the Act.

26. Resolved unanimously, That the thanks of this Association be given to James Parkinson, Esq. for having accepted the chair; and for the able manner in which he has conducted the business of the meeting.

27. Resolved unanimously, That the thanks of this meeting to the Chairman be advertised in the public papers.

28. Resolved unanimously, That the Report of the Committee, the state of the Funds, and the above Resolutions, be sent to the Medical Journals for insertion.

JAMES PARKINSON, Chairman.

---

Messrs. MAUVAGE and Co. Chemists at Paris, have invented a blister plaster, on silk, blue on one side and black on the other, which is admitted into the private pharmacy of the King, and which unites to a great degree of neatness the advantage of being as energetic as the ordinary blisters made with cantharides, without possessing the inconvenience of acting on the urinary passages. They have also invented a composition for a other plaster, likewise on silk, which is transparent, called *taffetas vegeto-epispastique*, purposely intended for the daily dressing of blisters, issues, &c.—*Moniteur.*

*Medical and Anatomical School, St Thomas's and Guy's Hospitals.*—The usual Lectures at these Hospitals will commence the beginning of October; viz. *At St. Thomas's*—Anatomy and the

Operations of Surgery, by Mr. Astley Cooper and Mr. Henry Cline; Principles and Practice of Surgery, by Mr. Astley Cooper. *At Guy's*—Practice of Medicine, by Dr. Curry and Dr. Cholmeley; Chemistry, by Dr. Marcet and Mr. Allen; Experimental Philosophy, by Mr. Allen; Theory of Medicine and Materia Medica, by Dr. Curry and Dr. Cholmeley; Midwifery and Diseases of Women and Children, by Dr Haighton; Physiology, or Laws of the Animal Œconomy, by Dr. Haighton; Structure and Diseases of the Teeth, by Mr. Fox.—N. B. These several Lectures, with those on Anatomy, and on the Principles and Practice of Surgery, given at the Theatre of St. Thomas's Hospital adjoining, are so arranged, that no two of them interfere in the hours of attendance; and the whole is calculated to form a complete Course of Medical and Chirurgical Instruction. Terms and other Particulars may be learnt from Mr. Stocker, Apothecary to Guy's Hospital.

*Medical School, St. Bartholomew's Hospital.*—The following Courses of Lectures will be delivered at this Hospital during the ensuing Winter, to commence on the 1st of October.—On the Theory and Practice of Medicine, by Dr. Hue; on Anatomy and Physiology, by Mr. Abernethy; on the Theory and Practice of Surgery, by Mr. Abernethy; on Chemistry and Materia Medica, by Dr Hue; on Midwifery, by Dr. Gooch; Practical Anatomy, with Demonstrations, by Mr. Stanley.—Further Particulars may be obtained by applying to Mr. Wheeler, Apothecary, at the hospital; or of Messrs. Anderson and Chase, Medical Booksellers, 40, West Smithfield.

*St. George's Medical, Chemical, and Chirurgical School.*—The Courses will commence in the first week of October; namely, 1st. On the Laws of the Animal Œconomy and the Practice of Physic, at No. 9, George Street, Hanover Square; by George Pearson, M. D. F. R. S. Senior Physician to St. George's Hospital, &c. 2d. On Therapeutics, with Materia Medica and Medical Jurisprudence; by W. T. Brande, F. R. S. Professor of the Royal Institution, and by George Pearson, M. D. &c. &c. 3d. On Chemistry, at the Royal Institution, by W. T. Brande, Professor of Chemisty, R. I. 4th. On Surgery, by B. C. Brodie, F. R. S. Assistant-Surgeon to St. George's Hospital. 5th. Sir Everard Home will give, as usual, Surgical Lectures gratuitously to the Pupils of the Hospital.

*Middlesex Hospital.*—Dr. P. M. Latham and Dr. Southey will begin their Lectures upon the Practice of Physic and the Materia Medica at this Hospital, in the first week of October next.

Dr. Adams will commence his Course of Lectures on the Institutes and Practice of Medicine early in the month of October, at his house, No. 17, Hatton Garden.

Dr. Clough, Physician Accoucheur to the St. Mary-le-bonne General Dispensary, the Endeavour, and Benevolent Institutions, &c. &c:; announces the re-commencement of his Winter Course of Lectures, on the Science and Practice of Midwifery, &c. at his house, No. 68, *Berner's Street*, early in October.—Timely notice will be issued in most Daily Papers.

Dr. CLUTTERBUCK, Physician to the General Dispensary, *Aldersgate Street*, will commence his Autumn Course of Lectures on the Theory and Practice of Physic, Materia Medica, and Chemistry, on Friday, October the 3d, at ten o'clock in the morning, at his house, No. 1, in the *Crescent*, *New Bridge Street;* where further Particulars may be had.—Pupils are admitted, as usual, to attend the Medical Practice of the Dispensary, and, when qualified, to visit the patients at home.—Clinical Lectures, on the most interesting and instructive Cases that occur, will be given by the Physicians in rotation.

Mr. GOOD will commence his Course of Lectures on Nosology, Medical Nomenclature, and the Theory and Practice of Medicine, on Monday September 29, 1817, at the Crown and Rolls Rooms, Chancery Lane. The Course will extend to rather more than three months, and be repeated three times a year. From the comprehensiveness of the subject, a Lecture will be given daily, instead of every other day, as is the usual practice. The introductory Lecture will commence at half-past three o'clock in the afternoon; the subsequent Lectures at eight in the morning: the former will be open to the Medical Public, including Medical Pupils, by tickets, to be had gratuitously at any of the Medical Booksellers of the Metropolis, where the Terms for the Lectures may also be known.

Dr. MERRIMAN, Physician Accoucheur to the Middlesex Hospital, and Consulting Physician-Accoucheur to the Westminster General Dispensary, and Dr. LEY, Physician-Accoucheur to the Westminster Lying-in Hospital, will resume their Course of Lectures on the Theory and Practice of Midwifery and the Diseases of Women and Children, at the Middlesex Hospital, in the month of October.

Mr. JOHN TAUNTON, Member of the Royal College of Surgeons of London, Surgeon to the City, Finsbury, and Caledonian Dispensaries, and to the City of London Truss Society, will commence his Autumnal Course of Lectures on Anatomy, Physiology, Pathology, and Surgery, on Saturday, October 4, 1817, at eight o'clock in the evening precisely, at his Theatre of Anatomy, Hatton Garden.—Particulars may be had by applying to Mr. Taunton, 87, Hatton Garden.

*Great Windmill Street.*—Mr. WILSON and Mr. BELL will commence their Lectures on Anatomy and Surgery on the 1st of October, at two o'clock. Mr. Bell will give a separate Course of Lectures on Surgery in the evening. The Anatomical Demonstrations will be given by Mr. SHAW, who will direct the Students in their Dissections.—A Clinical Lecture will be given every week at the Middlesex Hospital.

Mr. Wadd, of Park Place, St. James's, is appointed Surgeon Extraordinary to His Royal Highness the Prince of Wales.

Dr. Adams is elected Corresponding Member of the Faculty of Medicine at Paris.

In the Press, and in six weeks will be published, in one closely printed volume octavo, about 300 pages, An Essay on the Prolongation of Life, and Conservation of Health; unfolding original Views and fundamental Principles for their attainment, and embracing Observations on the Nature, Cause, and Treatment of some of the principal Diseases which assail the British Constitution in its native Climate. To which is added an Appendix, containing Practical Researches on the Pathology, Treatment and Prevention of Gout, in all its Proteian forms: translated and condensed from the French of M. M. Guilbert and Hallé (just published), with Notes. By James Johnson, M. D. Surgeon to his Royal Highness the Duke of Clarence; author of the Influence of Tropical Climates on European Constitutions; and one of the Editors of the Medico-Chirurgical Journal and Review.

In the Press, a work entitled, "An Analysis of the Medicinal Waters in Llandrindod, in Radnorshire; by Mr. Richard Williams; dedicated, by Permission, to Her Royal Highness the Princess Charlotte.

Mr. Bailey, of Harwich, is preparing for the Press, "Observations on the Use of Belladonna, illustrated by some striking examples of its efficacy in painful disorders of the face."

Deaths.—George Hoggart Toulmin, M. D. of Wolverhampton, and author of the "Elements of the Practice of Medicine." —Mr. Sutton, Surgeon, Yoxall, Staffordshire.—Drowned while bathing, between Leith and Porto-Bello, Stephen Wright, M. D. aged 21.—At Inverary, Mr. George Pirrie, Surgeon.

